# Post-mortem MR angiography: quantitative investigation and intravascular retention of perfusates in ex situ porcine hearts

**DOI:** 10.1007/s00414-017-1763-7

**Published:** 2018-01-18

**Authors:** Bridgette Webb, Thomas Widek, Sylvia Scheicher, Thorsten Schwark, Rudolf Stollberger

**Affiliations:** 1 Ludwig Boltzmann Institute for Clinical Forensic Imaging, Graz, Austria; 2 Medical University of Graz, Institute of Forensic Medicine, Graz, Austria; 3 Institute of Medical Engineering, Graz University of Technology, Graz, Austria; 4 BioTechMed, Graz, Austria

**Keywords:** Post-mortem MRI, Angiography, Extravasation, Vessel permeability

## Abstract

As the implementation of minimally invasive imaging techniques in both forensic and pathological practice increases, research in this area focuses on addressing recognised diagnostic weaknesses of current approaches. Assessment of sudden cardiac death (SCD) can be considered one such area in which post-mortem imaging still shows diagnostic weaknesses. We hypothesise that magnetic resonance imaging (MRI) with an angiographic adjunct may improve the visualisation and interpretation of cardiac pathologies in a post-mortem setting. To systematically investigate this hypothesis, selected perfusates (paraffin oil, Gadovist®;-doped physiological solution and polyethylene glycol (PEG)) were injected into the left anterior descending (LAD) artery of ex situ porcine hearts to assess the visualisation of perfusates in MRI as well as their intravascular retention over 12 h. Morphological images were acquired and quantitative T_1_ maps were generated from inversion recovery data. Visualisation of vascular structure and image quality were assessed using signal-to-noise and contrast-to-noise ratios. Intravascular retention was assessed both visually and statistically using a volume of interest (VOI) approach to analyse significant changes in signal intensity in and around the filled LAD artery, as well as changes in the longitudinal relaxation time (T_1_) in adjacent myocardium. In addition to presenting possible mechanisms explaining perfusate extravasation given the increased permeability of post-mortem vessels, the potential diagnostic consequences of this phenomenon and the importance of contrast stability and extended intravascular retention are discussed. In light of our findings and these considerations, paraffin oil emerged as the preferred perfusate for use in post-mortem MR angiography.

## Introduction

In both forensic and pathological practice, minimally invasive techniques such as computed tomography (CT), magnetic resonance imaging (MRI) and targeted biopsies have been implemented in an adjunctive function prior to the performance of conventional autopsies [1, 2]. In specific cases (e.g. paediatric and perinatal deaths [3] or disaster victim identification (DVI) [4]), minimally invasive techniques have additionally demonstrated potential in triage processes. Such processes provide a decision basis for the implementation of more invasive techniques with respect to the initial post-mortem imaging findings.

Research in post-mortem imaging increasingly focuses on addressing and improving specific, recognised diagnostic weaknesses of current approaches, especially with regard to suspected natural deaths. One such weakness is the assessment of sudden cardiac death (SCD). The need to define the role of post-mortem imaging in this field was acknowledged in a review of the current state of post-mortem imaging for cardiovascular pathologies [5].

Post-mortem assessment of ischaemic heart disease (IHD), the most common underlying cause of SCD, requires careful examination of both the coronary arteries, for stenosis and occlusions, and of the myocardium for signs of ischaemia [6]. We hypothesise that post-mortem MRI (PMMR), including an angiographic supplement, may lead to improved visualisation and interpretation of cardiac pathologies. This hypothesis is based on current applications of cardiac MRI in clinical practice which provide detailed information regarding soft tissue lesions and pathologies [7]. Initial experience with post-mortem MR angiography (PMMRA) has demonstrated its technical feasibility in a small cohort [8] and provided an example acquisition protocol for ex situ hearts using a lipophilic contrast agent mixture (paraffin oil and Angiofil®;) [9]. Additionally, recent work systematically investigated a broader range of liquids potentially suitable for use as perfusates in PMMRA [10]. In this work, viscosity, considered to significantly influence post-mortem extravasation of liquids, as well as intrinsic properties influencing MR signal and contrast, was characterised across a forensically relevant temperature range [10].

The importance of intravascular retention in post-mortem angiography has been highlighted in research regarding post-mortem CT angiography (PMCTA) [11]; however, time becomes an even greater concern in the context of PMMRA. Sequence parameters for PMMR can be adapted to take advantage of post-mortem circumstances (e.g. lack of motion and flow) and achieve very high spatial resolution. PMMR examinations can, as a result, become quite time intensive, especially when compared to CT procedures.

Therefore, it becomes essential that extravasation of PMMRA perfusates is minimal. In a post-mortem context, it can be assumed that the extent of active transport mechanisms is virtually non-existent; however, the effects of passive diffusion and the bulk flow of fluid through intercellular clefts [12], abundant in the endothelium of the vascular system, should not be underestimated. Since these mechanisms do not rely on the expansion of cellular energy, but rather depend on vascular permeability [13], post-mortem decay processes may even enhance such passive transport of molecules across vessel walls due to decreased vascular integrity. Such increased vascular permeability presents a major challenge for angiography-based imaging approaches and diagnosis of vascular pathologies in post-mortem investigations due to the increased unpredictability of extravasation. In recent years, factors influencing extravasation have been investigated in animal models [14, 15] during the development of a standardised protocol for multi-phase PMCTA [16]. This research indicates that careful consideration of properties such as lipophilicity and viscosity, which affect the behaviour of perfusates in the post-mortem vascular system [17], is required for reliable filling of post-mortem vessels and consequent interpretation of post-mortem angiography results.

The objective of this paper is to use ex situ porcine hearts to quantitatively investigate the visualisation and intravascular retention of three perfusates (paraffin oil, Gadovist®;-doped physiological solution and polyethylene glycol (PEG) 200) which have previously been categorised as suitable for PMMRA based on in vitro experiments and contrast simulations [10].

## Methods and materials

### Sample preparation

Porcine hearts (*n*= 9) from a local slaughterhouse were examined ex situ (post-mortem interval ≈ 12 h) using MRI. Pigs were approximately 120 days old and there were no discernible cardiac pathologies visible. Each of the three selected perfusates (paraffin oil, Gadovist®;-doped physiological solution (2 mmol/l) and polyethylene glycol (PEG) 200) was injected into the left anterior descending (LAD) arteries of ex situ porcine hearts. Injection was performed via the left coronary orifice, where a silicon Foley catheter (14FR, BARD®;) was inserted. A fast, low-resolution sequence (acquisition time: 16 s) was used to confirm vessel filling prior to commencing image acquisition as detailed below. This procedure was repeated three times (A, B, C) for each perfusate (3), resulting in a total of nine porcine hearts. Injection volumes are found in Table 1.

**Table 1:** Injection volumes for ex situ porcine hearts (A, B and C)

Perfusate	Volume_A_(ml)	Volume_B_(ml)	Volume_C_(ml)
Gadovist®; solution	2.8	2.8	9.0
Paraffin oil	2.8	5.0	3.0
PEG 200	3.8	2.8	3.0

### MRI acquisition

**Fig. 1:**
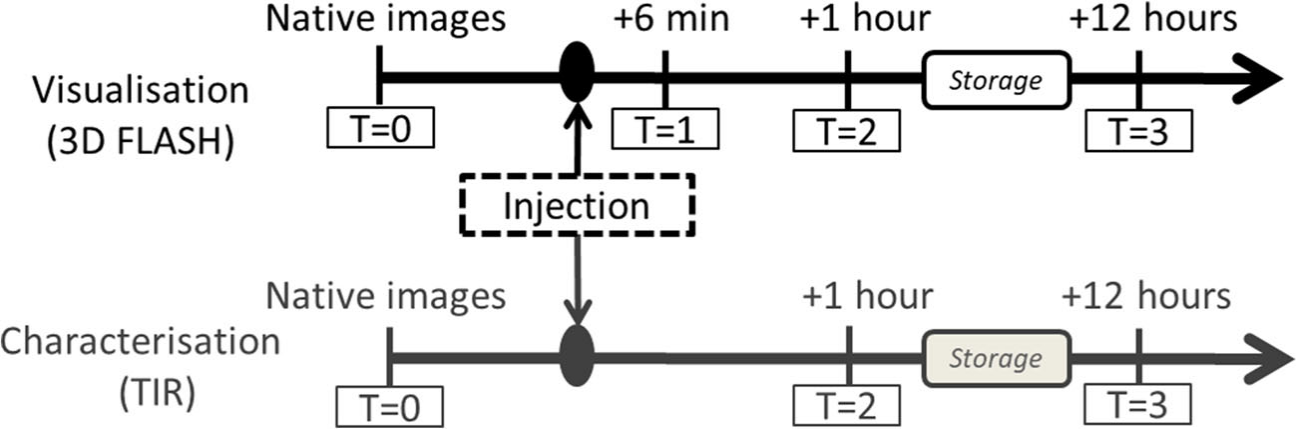
Scheme detailing perfusate injection and image acquisition. Quantitative data (TIR) was not acquired at *T*= 1 due to the duration of the sequence

MR images were acquired at 3 T (Skyra, Siemens Healthcare GmbH, Germany) using a 20-channel head coil (Siemens Healthcare GmbH, Germany) at the time points detailed in Fig. 1. Measurements were performed at room temperature and the temperature of the porcine hearts was monitored during examinations using a real-time fiber optic temperature sensor (Fluoroptic®;, LumaSense Technologies Inc, USA). For image acquisition, hearts were held in a glass container filled with flour. The temperature remained between 23 and 25 ^∘^C for each scan session. Between *T*= 2 and *T*= 3, hearts were stored at approximately 22 ^∘^C in a room adjacent to the scanner. For the visualisation of filled vessels, an adapted clinical sequence (3D FLASH; TR (ms)/ TE (ms)/FA (^∘^): 8/3.8/15, isotropic resolution: 0.8 mm) was used to cover the entire ex situ heart. The acquired images will hereafter be referred to as *morphological images*. Inversion recovery sequences (TIR; TR (ms)/TE (ms)/TI (ms): 10,000/9.7/ 50, 100, 200, 600, 1300, 2000, ETL: 8) were used to acquire data which was later fitted to generate T_1_ maps with a spatial resolution of 0.6×0.6 mm^2^ and slice thickness of 3 mm.

### Image processing and analysis

**Fig. 2:**
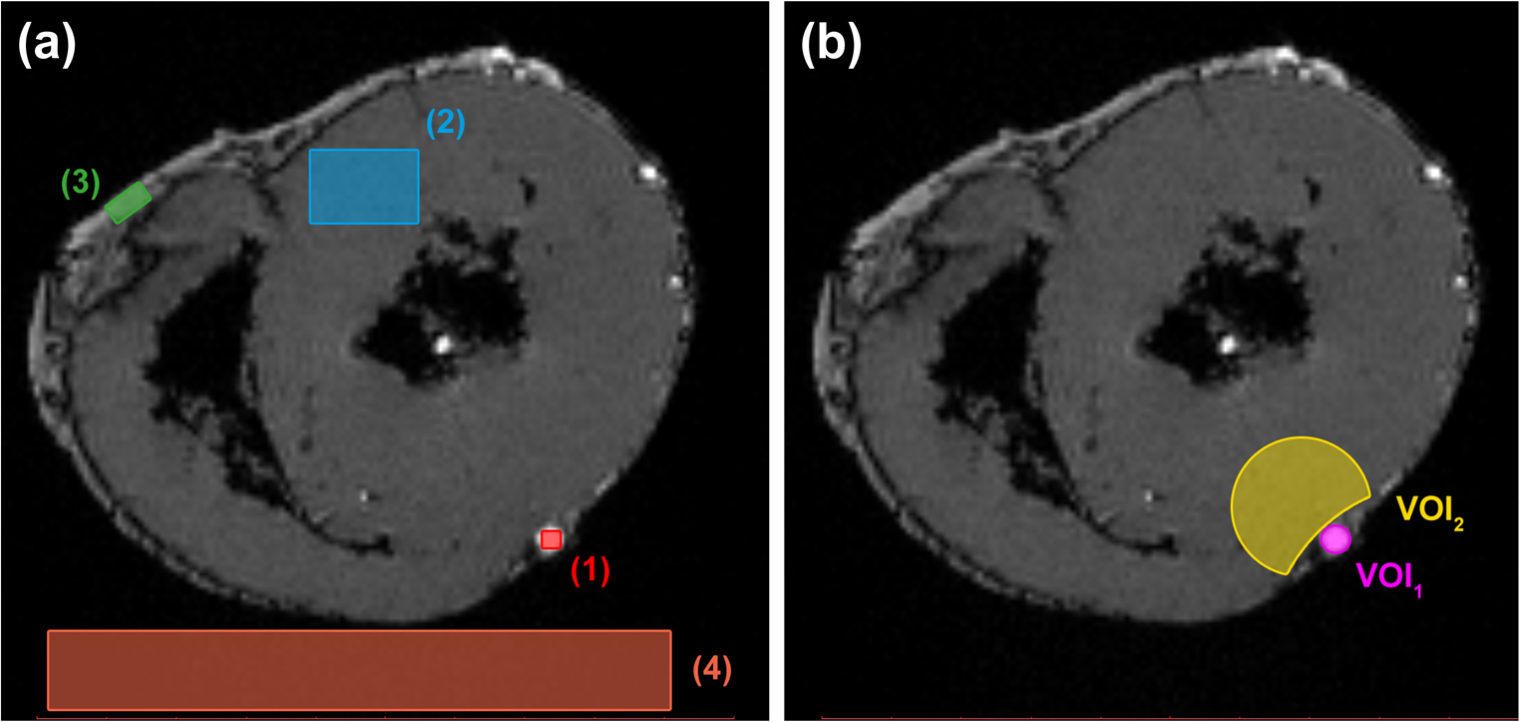
Morphological image displaying **a** ROIs for the calculation of SNR and CNR (1: perfusate, 2: myocardium, 3: epicardial fat, 4: noise) and **b** VOIs (single slice) for the LAD artery (VOI_1_) and adjacent myocardium (VOI_2_). VOI_*Q*_ took a similar form to VOI_2_. For orientation, the anterior wall of the heart is at the bottom of the image

In the post-injection morphological images, two volumes of interest (VOIs) were manually segmented using ITK-SNAP [18]. These VOIs corresponded to a segment of the filled LAD artery (VOI_1_), chosen to avoid filling artefacts (e.g. air), and adjacent myocardium (VOI_2_) in the same slices (Fig. 2). VOIs were defined in images acquired immediately following injection (e.g. VOI_1,2,*T*= 1_) and applied to images acquired 1 h later (e.g. VOI_1,2,*T*= 2_). Image registration was not performed as there was no change to the field of view (FOV) or movement of the sample during this time. VOIs were defined and used to describe signal intensity (SI) in the corresponding segments. SI values correspond to the brightness of each voxel in an image and are determined by a combination of tissue-specific properties and the selected MRI sequence parameters. VOIs were not applied to images acquired at *T*= 3, because the quality of the required image registration was not sufficient to enable direct application of the VOIs. An additional reason for not applying VOIs to the images acquired at *T*= 3 was that following the injection of certain perfusates (e.g. PEG 200), a reliable visual assessment of the vessels was no longer possible, potentially leading to uncertainty in the segmentation results.

The signal-to-noise ratio (SNR) and contrast-to-noise-ratio (CNR) were calculated for morphological images using a commercial software package (Matlab®; R2016) according to Dietrich et al. [19]. Both SNR and CNR are commonly used, quantitative measures of the ratio between the true signal (or true contrast) and background noise, the effects of which can, for example, be recognised as image graininess. These ratios were used in this work to compare different perfusates in images acquired under the same acquisition conditions. Regions of interest (ROI) for determination of SNR and CNR were defined in post-injection (*T*= 1) images corresponding to regions containing the following: perfusate, myocardium (remote), epicardial fat and noise (Fig. 2). This time point was selected as it offered the most reliable assessment of SNR and CNR prior to the influence of time and enabled a comparison of the perfusates under ideal (i.e. immediately following injection) conditions. Selection of a later time point may have negatively biased results due to the fast extravasation of certain perfusates.

Data acquired with the TIR sequences were segmented in a pre-processing step using a semi-automatic active contour segmentation in ITK-SNAP [18]. Segmentation was performed for a single inversion time (TI= 100) for each time point and applied to all TIR images for the respective time point. From segmented images, quantitative T_1_ maps were generated to visualise the voxel-wise mono-exponentially fitted (non-linear least squares) data. These images will hereafter be referred to as $T_{1} maps$. Post-injection T_1_ maps at various time points were registered to their native counterparts using a commercial software package (*imregister*, Matlab®; R2016). A single VOI (VOI_Q_) encompassing myocardium adjacent to the filled vessel, but excluding epicardial fat and the vessel itself, was defined in native T_1_ maps and applied to the registered images corresponding to later points in time.

All further image analysis was performed in Python [20]. Time-related changes to SI were analysed in the LAD artery (VOI_1_) and in adjacent myocardium (VOI_2_). Boxplots were generated to display changes in these VOIs over time. The VOI_Q_ was used to further characterise changes in the myocardium adjacent to the filled vessel during the first 12 h following injection. Histograms corresponding to the *T*_1_ relaxation times of the VOI_Q_ at each time point (VOI_Q, *T*= 0_, VOI_Q, *T*= 2_ and VOI_Q, *T*= 3_) were generated to display changes over time.

### Statistical analysis

To investigate whether SI in a given VOI changed between time points, one-way analysis of variance (ANOVA) on the entire corresponding VOI (all slices) was performed in Python [20]. The null hypothesis, that sample means were equal, was rejected for *p* values $<$ 0.05.

## Results

### Visualisation of vascular structure: SNR and CNR

**Fig. 3:**
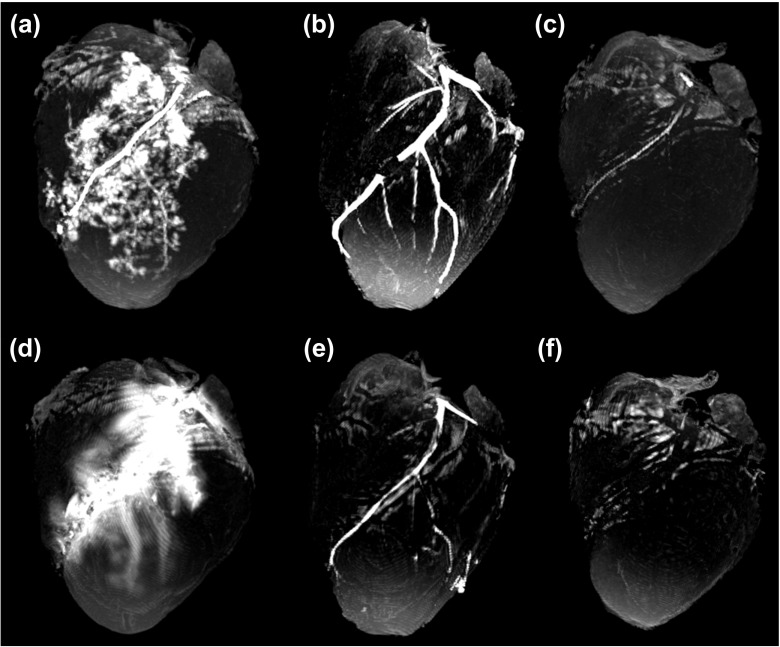
Maximum intensity projection (MIP) of example morphological images. Immediately after injection of **a** Gadovist®; solution, **b** paraffin oil and **c** PEG 200, and 12 h after injection of **d** Gadovist®; solution, **e** paraffin oil and **f** PEG200

In morphological images, all perfusates appeared hyperintense with the highest SI observed in voxels corresponding to the injected perfusate (calculated at *T*= 1). All perfusates demonstrated excellent contrast with surrounding tissue (Fig. 3). Myocardium SNR (mean ± standard deviation (SD)) was 34.9 $\pm $ 1.0 (range 34.05–36.26). In epicardial fat, SNR was 37.2 $\pm $ 1.6 (range 35.06–39.18). SNR values for each of the perfusates are displayed in Table 2. Of the three investigated perfusates, the Gadovist®; solution delivered the highest CNR against both myocardium (44.9 ± 3.4) and epicardial fat (43.6 ± 3.8) (Table 3).

**Table 2:** Signal-to-noise ratio (SNR) for all samples at *T*= 1

	Myocardium	Epicardial fat	Perfusate
Paraffin oil_A_	35.11	37.78	41.39
Paraffin oil_B_	35.04	36.24	45.35
Paraffin oil_C_	34.90	36.39	44.22
Mean $\pm $ SD	−	$-$	43.7 $\pm $ 2.0
PEG 200_A_	36.26	38.75	42.86
PEG 200_B_	34.05	35.06	39.70
PEG 200_C_	34.61	37.80	39.17
Mean $\pm $ SD	−	−	40.6 $\pm $ 2.0
Gadovist®; solution_A_	34.59	37.26	44.34
Gadovist®; solution_B_	34.43	36.11	48.50
Gadovist®; solution_C_	34.86	39.18	48.97
Mean $\pm $ SD	$-$	$-$	47.3 $\pm $ 2.5
Mean $\pm $ SD	34.9 $\pm $ 1.0	37.2 $\pm $ 1.6	$-$

**Table 3:** Contrast-to-noise ratio (CNR) for all samples at *T*= 1

Perfusate	Myocardium	Epicardial fat
	(mean $\pm $ SD)	(mean $\pm $ SD)
Paraffin oil	39.5 $\pm $ 3.4	37.8 $\pm $ 5.1
PEG 200	34.0 $\pm $ 3.1	29.6 $\pm $ 6.3
Gadovist®; solution	44.9 $\pm $ 3.4	43.6 $\pm $ 3.8

### Intravascular retention of perfusates in the LAD artery over time and effect on adjacent myocardium

The time-dependent intravascular retention of the investigated perfusates was obvious upon visual inspection of the morphological images. This dependence was assessed by analysing both morphological images, SI in the vessel (VOI_1_) and in surrounding myocardium (VOI_2_), and T_1_ maps, where the relaxation behaviour of myocardium adjacent to the filled vessel (VOI_Q_) was characterised. Table 4 summarises the ANOVA results for the relevant VOIs.

**Table 4:** *p* values from the one-way analysis of variance (ANOVA) for changes in mean SI in VOI_1_ and VOI_2_ as well as for changes in mean *T*_1_ values in VOI_Q_

	*VOI*_1_ (*T* = 1 *vs*.*T* = 2)	*VOI*_2_ (*T* = 1 *vs*.*T* = 2)	*VOI*_*Q*_ (*T* = 0 *vs*.*T* = 2)	*VOI*_*Q*_ (*T* = 2 *vs*.*T* = 3)
Paraffin oil_A_	0.067	0.724	0.056	0.227
Paraffin oil_B_	0.016	0.066	0.765	0.763
Paraffin oil_C_	0.116	0.070	0.637	0.780
PEG 200_A_	≪ 0.001	≪ 0.001	0.568	0.826
PEG 200_B_	≪ 0.001	0.040	0.812	0.001
PEG 200_C_	≪ 0.001	≪ 0.001	0.333	0.008
Gadovist®; solution_A_	0.297	≪ 0.001	≪ 0.001	≪ 0.001
Gadovist®; solution_B_	0.036	≪ 0.001	≪ 0.001	≪ 0.001
Gadovist®; solution_C_	≪ 0.001	≪ 0.001	≪ 0.001	≪ 0.001

#### Changes to SI in the LAD artery (VOI_1_) during the first hour after injection (*morphological images*)

**Fig. 4:**
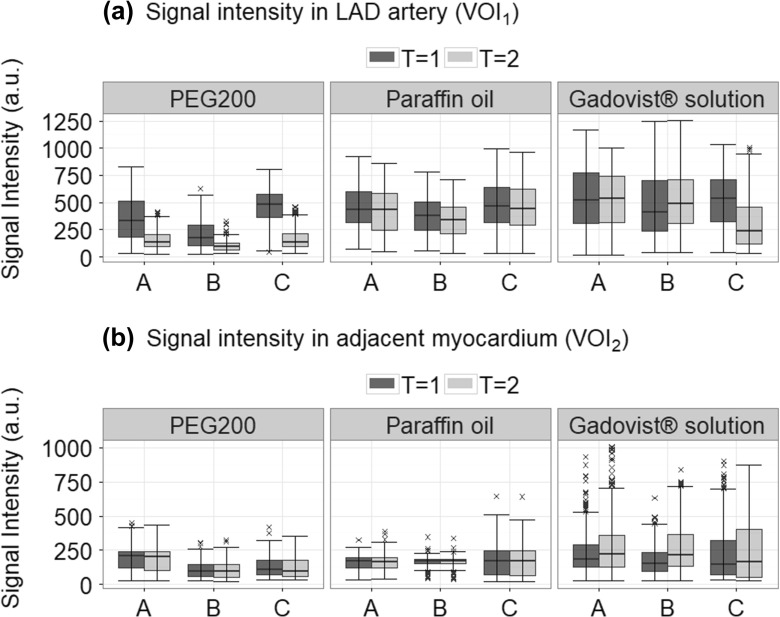
Boxplots representing signal intensity (SI) in **a** left anterior descending (LAD) artery (VOI_1_) immediately following injection (*T*= 1) and 1 h after injection (*T*= 2) and in **b** myocardium adjacent to the filled LAD (VOI_2_) immediately following injection (*T*= 1) and 1 h after injection (*T*= 2). A, B and C represent the three repetitions

Figure 4a demonstrates that the SI of paraffin oil in the LAD artery only minimally decreased in the first hour after injection. ANOVA of VOI_1_ indicated that this change was only statistically significant in one case (paraffin oil_B_). In porcine hearts injected with PEG 200, a decrease in SI was observed for all samples. This corresponded to visual observations, which indicated the disappearance of PEG 200 within the first hour. Changes to SI in these samples were all found to be statistically significant. For Gadovist®;filled arteries, both an increase (Gadovist®;_B_, statistically significant) and decrease (two samples, in Gadovist®;_C_ statistically significant) in SI were observed during the first hour.

#### Changes to SI in adjacent myocardium (VOI_2_) during the first hour after injection (*morphological images*)

The same analysis (Fig. 4b) was also performed for VOI_2_. Following ANOVA, no evidence of statistically different mean SI values was observed for paraffin oil within the first hour following injection. In PEG 200 samples, minimal differences over time were seen in all samples. ANOVA additionally found all these differences to be statistically significant. All Gadovist®; samples demonstrated an increase in SI within the first hour following injection. The statistical significance of these observations was also supported by the ANOVA results.

#### Changes in *T*_1_ of adjacent myocardium during the first 12 h after injection (T_1_ maps)

**Fig. 5:**
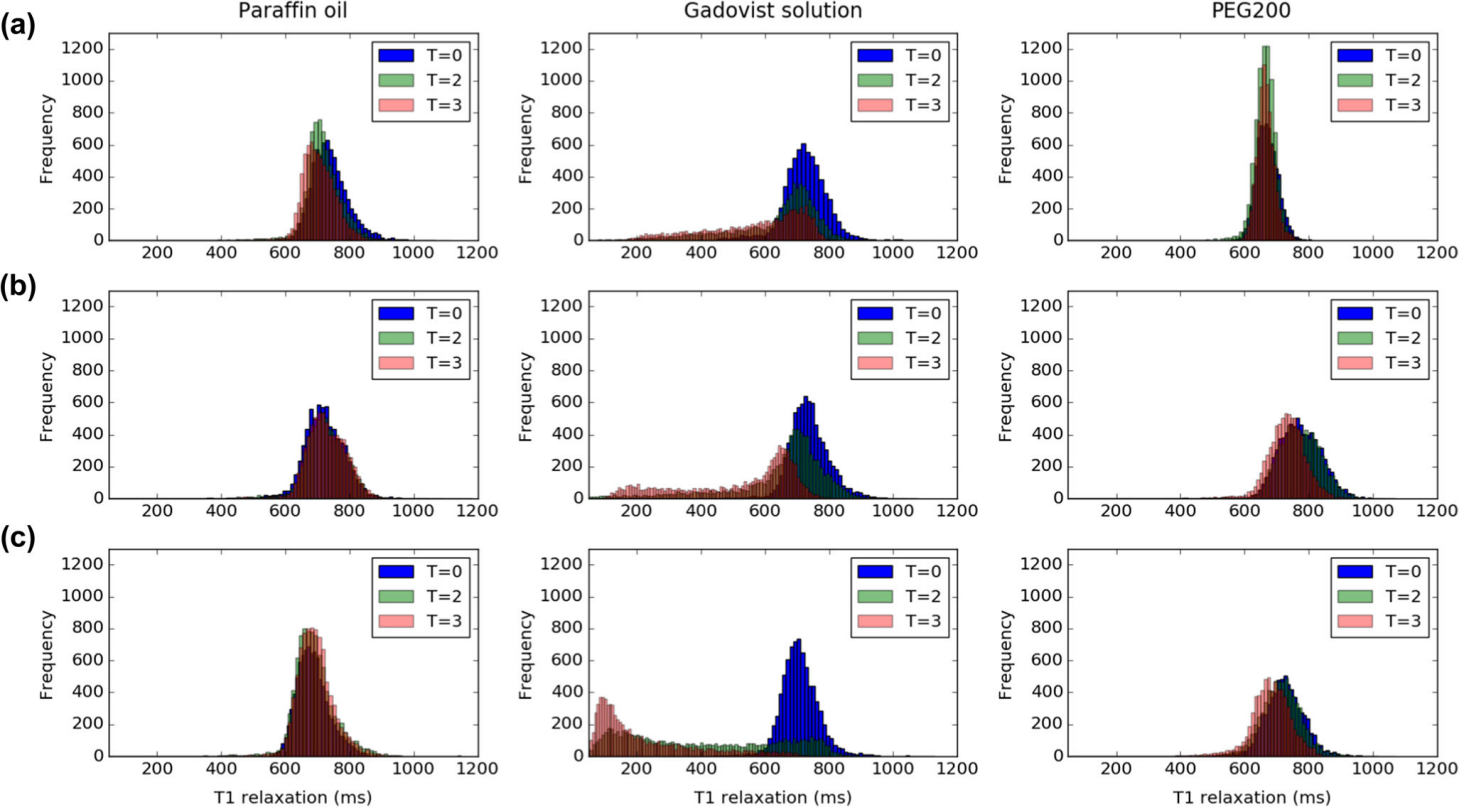
Distribution of *T*_1_ values (A, B, C) in myocardium adjacent to the left anterior descending artery (LAD) prior to perfusate injection (*T*= 0), one (*T*= 2) and 12 h (*T*= 3) later

To characterise changes in myocardium adjacent to filled vessels in more detail, T_1_ maps were analysed at three time points. Figure 5 shows the distribution of *T*_1_ values corresponding to VOI_Q_ at each of the measured time points. While *T*_1_ remained constant for most voxels where paraffin oil or PEG 200 was injected, the distribution of *T*_1_ values distinctly move to the left following injection of the Gadovist®; solution, indicating a shortening of *T*_1_. Within the first hour, ANOVA indicated no evidence of statistically significant changes in mean *T*_1_ values in myocardium for samples injected with paraffin oil and PEG200. Within the same time frame, all samples containing the Gadovist®; solution displayed statistically significant changes in *T*_1_. For data acquired after 1 and 12 h, samples with Gadovist®; solution as well as two additional PEG 200 samples revealed statistically significant changes in *T*_1_ values of myocardium adjacent to the filled LAD artery.

## Discussion

### Visualisation of vascular structure

SNR provided an indication of image quality and a measurement of SI taking into account the noise present in the image. Additionally, contrast between a particular region (or in practice a pathology) and surrounding tissue, once again taking into account the noise present in the image, was described by CNR. All morphological images were acquired with the same sequence and SNR in remote myocardium and epicardial fat remained constant across all samples (Table 2). Therefore, CNR was deemed a valid means to compare the contrast achievable using each of the investigated perfusates. The sequence used to acquire the morphological images was *T*_1_-weighted (*T*_1_w); therefore, it was expected that the shorter the *T*_1_ relaxation of a given perfusate, the brighter the vessels would appear. This behaviour was confirmed experimentally, with the Gadovist®; solution (*T*_1_[23 ^∘^C]: 100 ms) [21] displaying the highest CNR, followed by paraffin oil (*T*_1_ [23 ^∘^C]: 207 ms) [10] and PEG 200 (T_1_ [23 ^∘^C]: 216 ms) [10]. Nevertheless, it should be noted that although differences were observed between the perfusates in terms of CNR, all vessels filled with any of the investigated perfusates displayed excellent contrast with surrounding tissue, be that myocardium or epicardial fat. Additionally, due to the intensity of the vessels, maximum intensity projection (MIP) and volume rendering techniques were successfully applied (Fig. 3).

### Temporal variations of intravascular retention in the LAD artery

Whether perfusates escaped the LAD artery and the rate at which such extravasation occurred varied depending on the perfusate. Various models exist which attempt to describe the regulation of vascular permeability and the transport of molecules across the vascular barrier, with the exact mechanism describing transport out of the lumen and into surrounding tissue depending on vessel type, organ, kinetics of transport and the nature of the substance being transported [22]. These mechanisms have primarily been investigated in living systems; nevertheless, some aspects may be transferable to a post-mortem context. Extravasation of perfusates from the vessel into which they were injected can be described by at least two mechanisms. The first involves the passive transport of small molecules across the vessel wall. Even in living systems, suitable molecules can extravasate spontaneously via this mechanism [22]. In this case, both molecular (e.g. size, polarity, hydrophilicity and viscosity) and vascular properties play an important role in determining which substances permeate the vascular wall. The in vivo behaviour of gadolinium chelates commonly used in contrast-enhanced MRA (CE-MRA), which penetrate vessel walls to enhance tissue signal [23], provide an example of such permeation. Since vessel integrity is assumed to decrease post-mortem compared to in vivo conditions, it logically follows that the physiological solution containing Gadovist®; quickly diffused into adjacent myocardium. The same mechanism may also explain the disappearance of PEG 200 from the LAD artery within the first hour after injection and its effects on surrounding myocardium in the time thereafter. Similarities in the molecular properties influencing permeability of the vessel wall can be found between PEG 200 and Gadovist®; molecules. For example, both molecules can be classified as polar and are highly soluble in aqueous environments. Conversely, paraffin oil is non-polar and virtually insoluble in aqueous environments, indicating that the passive transport of these molecules may have been inherently positioned to fail.

The second mechanism which may explain extravasation involves penetration of the capillary network, followed by extravasation across the much more permeable single-layer epithelium of the capillaries. Extravasation of PEG 200 and Gadovist®; into surrounding tissue via this mechanism is also plausible. Regarding paraffin oil, a study by Grabherr et al. [15] described the viscosity of a lipophilic contrast agent as a trigger of embolisation in post-mortem capillaries, thus directly determining the calibre of vessels able to be filled. This work supports the hypothesis that paraffin oil remained intravascular due to its inability to penetrate the microcapillaries, therefore being unable to take advantage of the increased permeability of these vessels.

### Potential diagnostic consequences of extravasation

In addition to temporal variations between perfusates, the effects of displacement on the resulting MR images and diagnosis possibilities also varied.

As indicated by CNR, the Gadovist®; solution, which quickly diffused into small vessels and adjacent myocardium, generated excellent contrast with both remote myocardium and epicardial fat. Furthermore, immediately following injection, many small, filled vessels were observed in detail in the T_1_w images (Fig. 3a). However, after the first hour, contrast between the LAD artery and adjacent myocardium began to diminish due to the propagation of the solution further into surrounding tissue. This had the effect of reducing discrimination between vessels and myocardium, an effect which intensified with time and which can be seen in images acquired 12 h after injection. In Fig. 3d, the LAD artery seemed to have entirely disappeared. One could argue that an enhancement effect, as seen in clinical late gadolinium enhancement (LGE), would be beneficial in the post-mortem detection of myocardial infarction. However, this study demonstrated that even in non-pathological porcine hearts, distribution of the Gadovist®; solution was fast and unpredictable. Nevertheless, Gadovist®; solution as a perfusate may contribute to additional enhancement effects in regions corresponding to specific pathologies. Unfortunately, this intriguing aspect fell outside the scope of this work.

Paraffin oil, which primarily remained intravascular, provided consistent visualisation of vascular morphology. Analogue to PMCTA, which visualises vascular occlusions and morphology, T_1_w imaging, visualised paraffin oil filled vessels without confounding signal from epicardial fat or myocardium. The added value of PMMRA lies however not in its ability to closely mimic images acquirable in PMCTA, but in its potential to assist in the characterisation of a suspected vascular occlusion. When a filled vessel abruptly ceases, the burning question is why? Through its superior ability to distinguish subtle differences in soft tissue, MRI may offer advantages in the assessment of occlusions compared with CT and provide additional information to address this question.

PEG 200 offered neither extended intravascular retention nor enhancement effects such as those observed following injection of the Gadovist®; solution. PEG 200 appeared hyperintense in the filled LAD arteries in morphological images acquired immediately after injection; however, within an hour of injection, the solution was no longer visible. The slight increase in the *T*_1_ values in VOI_Q_ in two samples between the last two measurement time points (i.e. 1 and 12 h) supports the hypothesis that PEG 200 escaped into surrounding myocardium during this time. Unlike the Gadovist®; solution, which strongly shortened the longitudinal relaxation times of protons in surrounding water molecules, the slightly shortened *T*_1_ relaxation times observed in the case of PEG 200 were most likely a direct measurement of the protons within the –CH_2_ bonds [24] of the PEG molecule, thereby explaining the much milder effect. Since the effects of PEG 200 extravasation cannot be considered an improvement for diagnostic purposes, this solution should be disregarded as a possible perfusate for PMMRA.

### Limitations

Limitations in this study include the non-standardised injection of the perfusate. Although injection was performed by the same person in each case, the pressure of the injections was not measured as the porcine hearts needed to be kept in the same position in the scanner suite before and after injection. Furthermore, there were differences in the injection volumes (Table 1). This was due to placement of the catheter used to inject perfusates into the LAD artery. In some cases, perfusate escaped into the left ventricle, meaning more perfusate had to be injected to fill the LAD artery. The probability of leakage via this mechanism after the artery was filled was minimised by occluding the vessel at its origin in the aorta with a small plastic stopper. As previously mentioned, a fast, low-resolution sequence (acquisition time: 16 s) was used to confirm vessel filling prior to commencing the sequences used for analysis in this work. The findings in this study should be regarded with the number of examinations in mind. A total of nine porcine hearts were examined, corresponding to three hearts (A, B, C) for each perfusate. Through these repetitions, the authors sought to at least partially capture the variation. Nevertheless, observations across a larger sample size would no doubt improve the robustness of the underlying statistical analysis even more. Finally, since porcine hearts obtained from a local slaughterhouse were used, the investigation of perfusates in the presence of known pathologies was not possible. This would be of particular interest in the case of the Gadovist®; solution, where additional enhancement corresponding to pathological regions may occur.

### Future research

The scope of the current study was limited to the examination of perfusates in non-pathological porcine hearts. The authors suggest that future research should seek to evaluate perfusates in controlled animal experiments to examine the behaviour of perfusates in the presence of known pathologies (e.g. early acute myocardial infarction). Thereafter, the in situ feasibility of PMMRA should be investigated using cases of suspected SCD. Finally, the diagnostic potential and added value of the technique needs to be systematically evaluated and compared with current imaging and autopsy practices.

## Conclusion

We hypothesise that post-mortem MR angiography may improve the radiological detection and interpretation of cardiovascular pathologies in post-mortem investigations. In this context, we investigated the visualisation and intravascular retention of three perfusates in ex situ porcine hearts to evaluate their suitability for use in PMMRA.

All investigated perfusates (paraffin oil, Gadovist®;-doped physiological solution and PEG 200) generated excellent contrast against post-mortem porcine myocardium and epicardial fat. Gadovist®; solution and PEG 200 quickly extravasated (within 1 h), while paraffin oil remained intravascular for the duration of examinations (12 h). Passive transport of molecules determined by factors such as size, polarity, hydrophilicity and viscosity across the post-mortem vessel wall (more permeable than in vivo) or via penetration of the capillary network were presented as a likely mechanism to explain the observed perfusate-dependent rates of extravasation.

Since PMMR is more time intensive compared to its CT equivalent, the rate of perfusate extravasation plays a crucial role when evaluating the suitability of perfusates for application in PMMRA. Diagnostic consequences of extravasation, such as reduced discrimination between vessels and adjacent tissue due to shortened *T*_1_ relaxation in such tissue and poor visibility of vascular structure due to perfusate disappearance, were observed for the Gadovist®; solution and PEG 200, respectively. Therefore, paraffin oil with its strong, stable contrast against surrounding tissue and extended intravascular retention was considered most suitable for use as a perfusate in PMMRA.
